# Pharmacy student’s awareness, perceptions, and supportive environment as determinants of their intent to pursue postgraduate education in pharmaceutical marketing: a cross-sectional study

**DOI:** 10.1186/s12909-026-09411-4

**Published:** 2026-05-13

**Authors:** Ahmed M. Ashour

**Affiliations:** Department of Pharmacology and Toxicology, College of Pharmacy, Umm Al-Qura University, Makkah, Saudi Arabia

**Keywords:** Postgraduate education, Pharmaceutical marketing, Awareness, Perceived Importance

## Abstract

**Introduction:**

The pharmacy industry faces a significant challenge in maintaining a qualified workforce within the marketing sector. The purpose of the current study was to quantitatively investigate the combined effect of level of awareness, perceived importance, and the presence of a supportive environment on the intent to pursue a master’s degree in pharmaceutical marketing among undergraduate pharmacy students.

**Methods:**

We conducted an institutional cross-sectional study among undergraduate pharmacy students at a large public university in Saudi Arabia. Questionnaires were distributed electronically and students were given five weeks to complete and return them via email. Data were collected between March 25, 2025, and April 29, 2025. The data was analyzed using the Statistical Package for Social Sciences (SPSS).

**Results:**

A total of 421 students completed and returned the questionnaires. More than 65% of participants reported a GPA between 3.1 and 4.0, and nearly half (48.4%) were aged between 21 and 23 years. There was a combined effect of awareness level, perceived importance, and availability of a supportive environment on their intent to pursue a master’s degree in pharmaceutical marketing (*p* < 0.001). Among these factors, perceived importance demonstrated the strongest independent influence (*p* < 0.001), surpassing the effects of awareness or supportive environment.

**Conclusions:**

The combined influence of awareness, perceived value, and supportive environments significantly impacts undergraduate pharmacy students’ intent to pursue higher education in pharmaceutical marketing. Strengthening awareness campaigns, enhancing the perceived value of advanced pharmaceutical marketing education, and providing supportive environments could help build a skilled workforce and bridge the current gap in pharmaceutical marketing expertise.

**Patient or public contribution:**

Patients or members of the public were not directly involved in the design, conduct, analysis, or interpretation of this study. This is because the research focused on undergraduate pharmacy students’ awareness, perceptions, and intentions regarding postgraduate education in pharmaceutical marketing, which is an educational and professional development topic rather than a patient-centered clinical issue. However, elements of perceived importance included consideration of potential impacts on patient outcomes, reflecting an indirect acknowledgment of patient benefit. Future research may incorporate patient or public perspectives to better understand how pharmaceutical marketing education influences healthcare delivery and patient care.

**Supplementary Information:**

The online version contains supplementary material available at 10.1186/s12909-026-09411-4.

## Introduction

The pharmacy industry remains an influential sector in promoting positive healthcare outcomes across the world. Professionals in different pharmaceutical areas play vital roles within the healthcare paradigm, from the dispensing of medication to creating awareness among patients and even other industry stakeholders with responsibility of ensuring safety of medications and procedures used in pharmacy [1, 2]. While approaches like on job training and organized seminars, as well as access to information related to pharmacy through the internet continue to equip pharmacy students and professionals with relevant knowledge towards efficient performance and execution of their duties within the pharmacy industry, formal education remains the most promising approach through which these professionals can become optimally prepared for service delivery within the pharmacy sector [3–5]. In particular, the marketing area in pharmacy is one primary section that calls for highly trained individuals capable of supporting innovative methods geared towards ensuring better healthcare from all spheres of the pharmaceutical industry. From promoting creative works to creating drug and medication awareness among patients and retailers, as well as the availing information to manufacturers on emerging trends that must be put into consideration during drug production, marketing professionals in the line of pharmacy prove inevitably important for the delivery of quality and safe services. Furthermore, recent shifts in the pharmaceutical labor market, research findings indicate, that marketing-related roles increasingly require advanced analytical and strategic skills which cannot be sufficiently attained through undergraduate training alone [5].

According to Alkhateeb et al., students successfully completing a master’s degree in pharmaceutical marketing are equipped with requisite knowledge, skills, and abilities that place these graduates in a better position to pursue managerial responsibilities within the wider pharmaceutical industry. Individuals equipped with the knowledge, skills, and abilities associated with the degree of pharmaceutical marketing prove vital in the pharmacy industry as they understand various elements related to the efficient operation of the pharmaceutical industry from managed care, regulation, ethics, and the management of the sales workforce [5]. These individuals are also fully informed and knowledgeable on organizational behavior, an incredibly important area for the successful operation of the pharmaceutical industry [6, 7]. Although postgraduate programs in pharmaceutical marketing have traditionally targeted professionals with prior industry experience, the growing complexity of pharmaceutical markets has served to create a new demand for early-career graduates who enter the workforce with specialized marketing competencies. Studies from various regions further highlight the widening gap between industry needs and the preparedness of entry-level pharmacy graduates, importantly suggesting that a lack of advanced training contributes to persistent inefficiencies in market research, patient outreach, and forecasting within the pharmaceutical sector.

Unfortunately, despite this critical significance of a qualified workforce within the pharmaceutical marketing, current research shows that there is reluctance and consequently minimal uptake of postgraduate education in pharmacy marketing. Yenet et al. support this position noting that due to lack of highly trained professionals in the pharmaceutical marketing area, many organizations, particularly those involved in the importation and distribution of drugs in many nations, lack requisite information as well as rely on poor market research, challenges that sometimes hinder their success in the pharmacy industry [8]. The problem of drug shortage experienced in many nations and especially during critical times like during pandemics as described by Shukar et al. is also another major indicator that players in the pharmacy industry fail to recognize such demand in time. This challenge is directly attributable to the lack of adequate professionals with sufficient knowledge in pharmaceutical marketing who can ensure timely identification of such needs and communicate the information to relevant players in a timely manner to help nations avoid such problematic drug shortages [9]. Further, in a study where the authors focused on understanding pertinent issues that affect the pharmaceutical supply chain, Singh et al. confirm that there is scarcity of research in pharmacy in many nations, a role that could well be accomplished through qualified marketing professionals in the pharmacy industry [10]. These trends collectively point to an unmet workforce need, emphasizing that encouraging pharmacy undergraduates to consider postgraduate specialization may help bridge emerging skill gaps before they enter the profession. The cumulative impact of these problems facing the pharmaceutical industry from a marketing perspective is an indication of a need for research that aims at understanding various aspects related to the attainment of master’s degree in pharmaceutical marketing, an achievement that can in a great way help in addressing many of the challenges pointed out in current literature within the pharmacy industry.

In this regard, our study aimed at understanding undergraduate pharmacy students’ level of awareness about pharmaceutical marketing postgraduate education, their perceived importance of such education from both the perspective of their career development, industry players, and the patients as well, and the existence of supportive environment have on the intent of such students towards pursuing a master’s degree in pharmaceutical marketing. In terms of the guiding model, it is vital to note here in this study we adopted the Theory of Planned Behavior (TPB) as the explicit intention-formation model where awareness maps to attitudes, support aligns with subjective norms, and supportive environment strengthens perceived behavioral control by reinforcing capability and expected benefits. We adopted this model because together, these constructs can predict intention which in turn drives behavior. In this context, *perceived importance* of pharmaceutical marketing is conceptualized as reflecting students’ attitudes toward the behavior, notably as it captures their evaluation of the relevance and value of pursuing postgraduate education in this field. On the other hand, supportive environment as explored in this study aligns with subjective norms, importantly as it reflects perceived social pressure and encouragement from peers, faculty, and professional networks. Lastly, awareness is linked to perceived behavioral control, as greater knowledge of educational pathways and career opportunities enhances students’ perceived ability to pursue postgraduate studies. This mapping provides a theoretically grounded framework for examining the determinants of students’ intentions. Ultimately, the model is suitable for this study in that while TPB traditionally emphasizes direct measures of attitudes, norms, and control, the use of context-specific proxy variables in this study allowed for a more applied assessment of these constructs within pharmaceutical education.

## Methods and materials

### Study design, location, and time

We conducted a cross-sectional survey among pharmacy students at a large public university in Saudi Arabia who were enrolled between the first and sixth years of the pharmacy program. We carried out the study from March 25, 2025 to April 29, 2025. This university was selected because it offers one of the largest accredited pharmacy programs in the region. Our primary goal was to investigate how the level of awareness, perceived importance, and the existence of a supportive environment impact the intention of undergraduate pharmacy students’ towards pursuing a master’s degree in pharmacy marketing.

### Inclusion and exclusion criteria

Upon receiving ethical approval from the Institutional Review Board of the study institution, we posted recruitment notices across campus inviting undergraduate pharmacy students enrolled between the first and sixth year of the program to participate in the study. We fully communicated the purpose of the study and the role of the participants, and requested those interested in volunteering in the study to show their intention by sending an email to the lead researcher for them to be provided with the study data collection tool. For any minors (students below 18 years of age), we obtained supporting authority from their parents and or guardians before allowing these minors to participate. In this regard, included participants were current students pursuing the Bachelor of Pharmacy degree program at the study institution. Students not in pharmacy or those in postgraduate program within the pharmacy area were excluded from the study.

### Recruitment and sample

As mentioned about, all students currently pursuing the Bachelor of Pharmacy degree program at the study institution were our target as the study population. The study employed convenience sampling method to recruit students who communicated their intention to participate in the study as requested in the notice we put within the university.

### Study variables

The study was guided by three multi-factor independent variables; pharmacy students’ awareness about post graduate pharmaceutical marketing, pharmacy students’ perceived value of postgraduate pharmaceutical marketing, and the existence of a supportive environment towards the pursuit of a master’s degree in pharmaceutical marketing. The awareness independent variable combined two sub-variables or factors, students previous formal training in pharmacy and their having of information about pharmaceutical marketing post graduate information. This variable was designed to capture both the depth and accessibility of information available to students, as awareness has been shown to strongly influence postgraduate decision-making in health sciences fields. Similarly, the perceived importance independent variable was made up of the students’ indication of how pharmacy marketing information at the postgraduate level would impact their career, and how it would impact healthcare outcomes for patients. This construct was included to assess not only personal career expectations but also students’ understanding of the broader professional and societal value associated with advanced pharmaceutical marketing competencies. The supportive environment was a complex variable made up of the existence of availability of scholarships, tuition assistance to offset cost of the program, flexible work schedule, and availability of flexible study programs such as accelerated master’s degree program or availability of fully online study programs. These factors were selected because institutional and financial support mechanisms are consistently identified in the literature as critical determinants of postgraduate enrollment among healthcare students. Students’ reported level of interest and motivation for pursuing master’s degree in pharmacy marketing formed the dependent variable of our study, intent. This intent variable functioned as the main outcome, reflecting students’ readiness and likelihood to translate awareness and perceived value into actual postgraduate pursuit. Our questionnaire here employed a limited number of items, including some binary measures in order to ensure feasibility and response completion. However, we note that this approach may have the overall impact of reducing the sensitivity of the instrument in capturing complex behavioral constructs such as attitudes and perceived control and additionally, some items may have been directive in nature, potentially influencing response patterns. These limitations were considered when interpreting the findings to ensure the proper conclusions from the results.

### Data collection tool and process

An invitation email and campus notice were distributed to all undergraduate pharmacy students (*N* = 648). In preparing the survey questionnaire, our process was informed by previous studies as well as current literature to pinpoint the critical issues for inclusion in the data collection items. In line with our study variables, we collected data on students’ reported level of motivation and interest to pursue a master’s degree in pharmacy marketing, their having or not having information about postgraduate pharmaceutical marketing education, their having of previous training in pharmacy or related area, their perceived level of importance of master’s degree in their career and how it might impact healthcare outcomes for patients, and their rating the importance of availability of supportive environment factors (scholarships, tuition assistance to offset cost of the program, flexible work schedule, and availability of flexible study programs such as accelerated master’s degree program or availability of fully online study programs) towards positively impacting their decision to pursue master’s education in pharmaceutical marketing. Two faculty members from the College of Pharmacy at the study institution reviewed the questionnaire for content validity. We used a sample of five pharmacy faculty members from the study institution to pilot test the questionnaire for appropriateness on elements like suitability of the questions, time needed to fill it by participants, and clarity of all included information. We emailed the structured questionnaire with 28 items to students responding to our notice about the study for the collection of the data. We received fully filled questionnaires from 421 students. The study was conducted within a single institution to allow for controlled data collection and consistency in educational exposure among participants. The questionnaire used in this study was developed specifically for the current research based on a review of relevant literature on postgraduate education intentions among pharmacy students. The instrument underwent content validation by two faculty members and pilot testing with five faculty members to ensure clarity, relevance, and appropriateness. The final English version of the questionnaire is provided as Supplementary File 1.

### Data quality assurance and analysis

We used the calculator by Raosoft (http://www.raosoft.com/samplesize.html*)* to ascertain the appropriateness of our sample, taking acceptable error (alpha) to be 5% and a 95% confidence interval. We organized the collected data, cleared it for erroneous entries or omissions, coded and as necessary transformed it to ready it for analysis. We used the Statistical Package for Social Studies (SPSS), version 25 to analyze the data, computing descriptive statistics for demographic variables and based on the Kolmogorov-Smirnov test for normality, adopted a ordinal linear regression modeling to understand how the study independent variables were impactful in influencing the intent by undergraduate students to pursue a master’s degree in pharmacy marketing. In terms of validity and reliability assessment, the exploratory nature of this study and the use of a newly developed instrument meant that detailed reliability and validity testing were limited, which represents a methodological limitation. Given the use of single-item and partially binary indicators, internal consistency measures such as Cronbach’s alpha were not appropriate for all constructs. This approach aligns with exploratory survey designs where constructs are operationalized using formative or proxy indicators rather than reflective multi-item scales. Future studies should employ validated multi-item scales to improve measurement robustness.

## Results

Upon distributing the survey to the target study population of the 597 students who indicated their intention to participate in the study, we received a total of 421 duly filled and free from error questionnaires. This indicated a response rate of 70.52%. Majority (65.8%) of the participants reported to have GPA of between 3.1 and 4, and most of them were in the age of 21 to 23 years (48.8%). About 54% of the participants (*N* = 226) of the participants were male. Further information about the demographic and other relevant attributes for the participants of this study are summarized in Table 1.

**Table 1:** General characteristics of the study participants

General Characteristics of the Participants		
Particulars	Frequency	Percent
Age		
< 18	2	0.5
18–20 years	159	37.8
21–23 years	180	42.8
24–26 years	75	17.8
> 26	5	1.2
Location of work		
In a city	389	92.4
In a village	32	7.6
GPA		
1.1 to 2	20	4.8
2.1 to 3	124	29.5
3.1 to 4	277	65.8
Satisfaction with their Education		
Very dissatisfied	10	2.4
Dissatisfied	52	12.4
Neutral	150	35.6
Satisfied	182	43.2
Very Satisfied	27	6.4
Marital status		
Married	6	1.5
Single	415	98.6
Nationality		
Non-Saudi	2	0.5
Saudi	419	99.5
Interest in Pharmaceutical Marketing Master’s Degree		
Yes	329	78.1
No	92	21.9

The ordinal linear regression model to check the impact of the different independent variables on the predicted variable, intent of pharmacy students to pursue a master’s degree in pharmaceutical marketing, produced the following results. We first tested for the normality of distribution of the variables using the Kolmogorov-Smirnov test, and found that all the variables were not normally distributed (*p* = 0.000 for all variables) as shown in Table 2 and the supporting details in Table 3 below.

**Table 2:** Tests of normality for study variables using Kolmogorov–Smirnov and Shapiro–Wilk tests

Tests of Normality						
	Kolmogorov-Smirnov^a^			Shapiro-Wilk		
	Statistic	df	Sig.	Statistic	df	Sig.
Intent	0.181	421	0.000	0.928	421	0.000
Awareness	0.192	421	0.000	0.908	421	0.000
PerceivedImportance	0.155	421	0.000	0.953	421	0.000
SupportiveEnvironment	0.138	421	0.000	0.931	421	0.000

a. Lilliefors Significance Correction

**Table 3:** Case processing summary for the dependent variable (students’ intention to pursue a master’s degree in pharmaceutical marketing)

Case Processing Summary			
		*N*	Marginal Percentage
Intent	0.67	1	0.2%
	1.00	43	10.2%
	1.33	11	2.6%
	1.50	1	0.2%
	1.67	67	15.9%
	2.00	74	17.6%
	2.33	124	29.5%
	2.50	2	0.5%
	2.67	51	12.1%
	3.00	47	11.2%
Valid		421	100.0%
Missing		0	
Total		421	

Captured in Tables 4 and 5, and 6, we produced a model fitting information to confirm the suitability of the model in predicting the dependent variable from the independent variables, and the model emerged to be a good fit (*p* = 0.000).

**Table 4:** Model Fitting Information for the Ordinal Logistic Regression Model

Model Fitting Information				
Model	-2 Log Likelihood	Chi-Square	df	Sig.
Intercept Only	1203.648			
Final	1161.264	42.384	3	0.000

Describes the comparison between the intercept-only model and the final model, including −2 log likelihood, chi-square, degrees of freedom, and significance level

Link function: Logit

**Table 5:** Goodness-of-Fit Statistics for the Ordinal Logistic Regression Model

Goodness-of-Fit			
	Chi-Square	df	Sig.
Pearson	2609.613	1878	0.000
Deviance	919.270	1878	1.000

Presents Pearson and Deviance chi-square tests used to assess how well the model fits the observed data

Link function: Logit

**Table 6:** Pseudo R-Square Values for the Ordinal Logistic Regression Model

Pseudo *R*-Square	
Cox and Snell	0.096
Nagelkerke	0.098
McFadden	0.027

Displays Cox and Snell, Nagelkerke, and McFadden pseudo R-square values indicating the explanatory power of the model

Link function: Logit

### Predicted model impact

It emerged that there is statistically significant impact of the fitted model comprising of the three independent variables; the level of awareness among the undergraduate pharmacy students, their perceptions regarding the importance of a master’s degree in pharmaceutical marketing, and the existence of a supportive environment towards the attainment of a master’s degree in pharmaceutical marketing. Overall, the model explained 9.8% of the variance in students’ intention to pursue a master’s degree (Table 6). At the individual level of the model, perceived importance of the attainment of a master’s degree in pharmacy marketing was the most impactful factor on the intention of undergraduate students towards pursuing a master’s degree in pharmaceutical marketing (*p* < 0.001). Both the level of awareness and existence of supportive environment were not statistically significant (p values of 0.692 and 0.206) respectively (Table 7).

**Table 7:** Parameter estimates from the ordinal logistic regression model showing the effect of awareness, perceived importance, and supportive environment on students’ intention to pursue a master’s degree in pharmaceutical marketing

Parameter Estimates								
		Estimate	Std. Error	Wald	df	Sig.	95% Confidence Interval	
							Lower Bound	Upper Bound
Threshold	[Intent= .67]	-3.772	1.199	9.897	1	0.002	-6.122	-1.422
	[Intent= 1.00]	0.204	0.671	0.093	1	0.761	-1.111	1.520
	[Intent= 1.33]	0.474	0.669	0.503	1	0.478	-.837	1.785
	[Intent= 1.50]	0.496	0.669	0.551	1	0.458	-.814	1.807
	[Intent= 1.67]	1.548	0.669	5.353	1	0.021	0.237	2.860
	[Intent= 2.00]	2.366	0.674	12.309	1	0.000	1.044	3.688
	[Intent= 2.33]	3.746	0.688	29.616	1	0.000	2.397	5.095
	[Intent= 2.50]	3.773	0.689	30.022	1	0.000	2.423	5.123
	[Intent= 2.67]	4.680	0.701	44.593	1	0.000	3.307	6.054
Location	Awareness	0.091	0.230	0.157	1	0.692	-.360	0.543
	PerceivedImportance	0.783	0.125	39.012	1	0.000	0.537	1.028
	SupportiveEnvironment	0.114	0.090	1.601	1	0.206	-.062	0.290

Linkfunction: Logit

## Discussion

Consistent with current literature, the regression model we developed predicts that the undergraduate student’s perceived importance postgraduate educational qualification in pharmaceutical marketing as an influential factor forming their intent towards attaining such qualification. The existence of a supportive environment and students’ level of awareness about pharmaceutical master’s degree education have an overall minimal impact of increasing their intent to want to pursue post graduate education in pharmaceutical marketing at the master’s degree level [11, 12]. These findings partially align with the statistical results of this study, where perceived importance emerged as the only statistically significant predictor of students’ intent, while awareness and supportive environment did not demonstrate significant independent associations. A concerted effort from all stakeholders is of incredible importance towards ensuring that the impact that these factors have are enhanced to help minimize the shortages within the pharmaceutical marketing area as current literature has pointed. However, it is paramount to note here that the current study did not measure workforce shortages directly, and therefore such implications should be interpreted as broader contextual observations rather than conclusions derived from the data. Notably, with increased intent among undergraduate pharmacy students to pursue master’s degree education, it is possible that in a couple of years from now the pharmacy industry will have plenty of qualified professionals who can support the efficient operation of this area, helping eliminate challenges as lack of information among key stakeholders within the drug supply chain, and ensure that there is proper projecting of required medications to curb shortages that are witnessed in different parts of the world [13, 14]. It is vital to point here that although these points are supported by existing literature, the present study only provides evidence on predictors of student intent, not on supply chain or global shortage outcomes.

The perceived importance of pursuing a master’s degree in pharmacy marketing emerged as a pivotal factor shaping the intent of undergraduate pharmacy students. This finding is evidenced by the strong positive regression coefficient associated with perceived importance in our model. The implication of this finding is that there is high level recognition among undergraduate pharmacy students of the significant benefits such a degree offers. Career advancement is one major area why many students would want to focus on attaining postgraduate education, and the findings of the current study support this notion as the perceived value of attaining a master’s degree in pharmaceutical marketing has the position to help professionals in the pharmacy industry be better positioned for managerial roles, consistent with current literature [6]. Besides its potential to enhance career prospects, an important attribute in the perceived importance of a master’s degree in pharmaceutical marketing is that it can improve patient health outcomes and streamline efficiency within the pharmaceutical supply chain especially when integrated with modern technology practices. Similar observations have been reported in studies from other regions, where pharmacy graduates viewed postgraduate marketing training as a pathway to both professional growth and improved sector performance [10, 15].

Notably, the emphasis placed on career advancement in a significant way highlights the pragmatic approach undergraduate pharmacy students take towards their education, viewing it as a strategic investment in their future professional success while at the same time recognizing that it can be beneficial to other players in the pharmaceutical industry. The acknowledgment of the degree’s potential to impact patient health outcomes as well as decision making by key players within the pharmaceutical industry is an indication of a growing awareness among students of the broader societal implications of their educational choices. Policymakers and other stakeholder’s, particularly those within the educational sector have the mandate to take note of the impact of the perceived importance of master’s degree education in pharmaceutical marketing as an indication that the recognizing of the role of pharmacy marketing in optimizing healthcare delivery is a demonstration that students show an understanding of the intersection between business acumen and healthcare efficacy. Accordingly, appropriate measures need to be put in place to support greater levels of perceived importance of the postgraduate master’s education in pharmaceutical marketing as one of the ways to reduce the shortage of qualified shortage that is cited in most of the current literature in this area [8]. Collectively, these findings demonstrate that the study successfully met its objective of assessing how students perceive the value of postgraduate pharmaceutical marketing education.

The availability of a supportive environment emerges as another useful but not necessarily statistically significant determinant influencing the intent of undergraduate pharmacy students towards pursuing a master’s degree in pharmaceutical marketing. Participants in this study indicate that the provision of accelerated programs, tuition stipends to alleviate financial burdens on the cost of post graduate education in pharmaceutical marketing, accommodative work schedules, as well as access to online classes collectively contribute to fostering an environment conducive to academic pursuit. The regression results confirm this, showing a positive but not statistically significant association between a supportive environment and student intent. The impact of such a supportive environment cannot be overlooked as a vital contributor of the general effect that the combined variables in the regression model for this study have on the intent and potentially the pursuit of a master’s degree in pharmaceutical marketing. Understandably, the availability of accelerated programs speaks to the desire for efficient educational pathways, catering to students’ aspirations for timely career entry [16–18]. The cost of pharmaceutical master’s degree education is a costly project that requires support for students to be able to achieve it [19, 20]. In addition, busy work schedules, especially within the pharmacy sector, prevent many students from wishing to pursue postgraduate education [21]. As a result, the provision of tuition stipends and accommodative work schedules addresses financial constraints and competing commitments, making education more accessible and feasible for a broader demographic of students. These measures can further be strengthened by the availability of online classes which are in line with the evolving landscape of education that are accommodating to diverse learning preferences and facilitating greater flexibility in scheduling. These findings are consistent with studies in health professions education that emphasize financial and institutional support as primary enablers of postgraduate enrollment [22].

The level of awareness regarding the master’s degree in pharmaceutical marketing is further an important factor in enhancing the intent by undergraduate pharmacy students in their efforts towards pursuing higher education in this field. Consistent with the current literature, the presence of information and awareness about the degree program significantly influences students’ perceptions and decision-making processes [23]. In the advanced technological environment of today access to information is highly important for decision making in any area, and it is no surprise that the level of awareness about a marketing master’s degree in pharmacy is one of the vital ingredients that may not only spark but ignite the desire and intent among undergraduate pharmacy students to pursue master’s degree education in this area [4]. The lack of awareness has the potential to impede students’ ability to fully comprehend the value proposition of the degree, in a significant way diminishing their inclination towards enrollment. Thus, stakeholders tasked with the responsibility of ensuring flow of information about such degree program within the education sector have to focus on creating increased awareness through targeted information dissemination efforts for the purpose of enhancing students’ understanding of the program’s relevance, benefits, and potential career trajectories. This finding is supported by our statistical analysis, where awareness contributed to the model but was not statistically significant in explaining students’ intent. Despite these valuable insights, several methodological limitations must be noted. The study was conducted at a single institution, which may limit the generalizability of the findings. Additionally, the use of self-reported measures introduces the possibility of response bias, and the cross-sectional design restricts the ability to infer causality between the independent variables and student intent. Overall, these factors are essential and while level of awareness and supportive environment do not emerge as statistically significant to the extent of the perceived importance of postgraduate education among pharmacy students, we note that although the model demonstrates low explanatory power (R² ≈ 0.098), these factors may contribute contextually to students’ intentions; however, only perceived importance demonstrated a statistically significant independent effect.

Critical to point here is that although the model explains approximately 9.8% of the variance in students’ intention to pursue postgraduate education, this level of explanatory power is not uncommon in behavioral and educational research, where intentions are influenced by a wide range of unobserved psychological, social, and contextual factors. The relatively low R² suggests that additional variable including but not limited to personal motivation, financial constraints, academic performance, career aspirations, and labor market perceptions, may also play a significant role. Furthermore, intention formation is inherently complex and may not be fully captured through a limited set of predictors. Therefore, while the identified factors are statistically significant, future research should incorporate a broader set of variables to improve model explanatory power. This finding is consistent with prior applications of TPB in educational contexts, where modest R² values are frequently reported due to the multifactorial nature of decision-making processes.

We noted here that this study, despite these important findings, has notable limitations. One, the reliance on data collected from a single institution is a major drawback here and future research can utilize data from multiple institutions to eliminate any potential bias. Also, the validation of the data collection tool was carried out by two staff members and substantiated through a pilot test of five individuals. We recommend that future research should be aware of this limitation and adopt other approaches towards validation of the data collection tool. The use of a single-institution sample limits the generalizability of the findings, as students may share similar educational experiences and institutional influences and in this regard, we acknowledge that these results may not be representative of pharmacy students in other settings or regions.

## Conclusion

The intent to pursue a master’s degree in pharmaceutical marketing is notably an important step towards starting such a process among undergraduate students in pharmacy. A variety of factors have the potential to impact this intent, accelerating the desire and the likelihood that more students end up pursuing such a degree. The findings indicate that perceived importance is the primary determinant of students’ intention, while awareness and supportive environment contribute contextually but do not demonstrate statistically significant independent effects. Ultimately, a cooperative effort from relevant stakeholders, from policy makers to key players within the educational sector, is necessary to enhance the impact that awareness, perceived value of importance of master’s education in pharmaceutical marketing, and a supportive environment have on the intent to pursue such education among undergraduate pharmacy students. The study provides evidence that perceived importance is a significant predictor of student intent, while awareness and supportive environment contribute contextually to students’ decision-making. These findings address the study objective of examining determinants of students’ intent. Future research should explore longitudinal outcomes to determine whether increased intent translates into actual enrollment and career progression in pharmaceutical marketing. Over time, such initiatives and efforts have the potential to help increase the number of pharmacy professionals with postgraduate training in the marketing sector of the pharmacy industry, further helping address critical challenges like lack of information among important players in the industry which comes with associated challenges as missed markets and drug shortage during critical times.

## Supplementary Information

Supplementary Material 1.

Supplementary Material 2.

## Data Availability

All data generated or analyzed during this study are included in this published article and its supplementary information files. Additional datasets used and/or analyzed during the current study are available from the corresponding author on reasonable request.

## Abbreviations

SPSS: Statistical Package for the Social Sciences
TPB: Theory of Planned Behavior
