# Supplementary material for: Pharmacy student’s awareness, perceptions, and supportive environment as determinants of their intent to pursue postgraduate education in pharmaceutical marketing: a cross-sectional study
Source: BMC Med Educ. 2026 May 13;26:1087. doi: 10.1186/s12909-026-09411-4 (PMC13340372; doi:10.1186/s12909-026-09411-4)
Supplement: Supplementary file 1 — Supplementary Material 1. [file 12909_2026_9411_MOESM1_ESM.pdf]

Kingdom of Saudi Arabia
Ministry of Education
Umm Al-Qura University
031

المملكة العربية السعودية
وزارة التعليم
جامعة أم القرى
٠٣١

Approval No. (HAPO-02-K-012-2025-01-2454)

FINAL APPROVAL FROM THE BIOMEDICAL RESEARCH ETHICS COMMITTEE

Principal Investigator: **Ahmed Mohammed Ashour**	Faculty: **Pharmacy**	Date: **02/01/2025**

Pharmacy Student's Awareness, Perceptions, and Supportive Environment as Determinants of their Intent to Pursue Postgraduate Education in Pharmaceutical Marketing: An Institutional Cross-Sectional Study

The Biomedical Research Ethics Committee has evaluated and examined the above-mentioned research proposal and has found it to be in accordance with the specifications and conditions of the ethics of scientific research.

The Committee has accordingly granted the Principal Investigator final approval concerning the ethics of scientific research

Principle Investigator is permitted to:

- Initiate the implementation of scientific research procedures within faculty facilities and laboratories, in addition to the regional research centers and hospitals
- Publish in scientific journals

Responsibility of Principal Investigator:

- Must provide a written statement to the Vice presidency of post-graduate studies and scientific research regarding any changes in the research plan, the committee shall decide whether a new approval is needed or not.

Director of Biomedical Ethics Committee

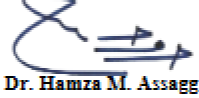

Dr. Hamza M. Assaggaf
Umm Al-Qura University

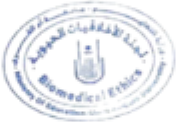

الرقم :   التاريخ : 02/01/2025   المرفقات :
