# Supplementary material for: Pharmacy student’s awareness, perceptions, and supportive environment as determinants of their intent to pursue postgraduate education in pharmaceutical marketing: a cross-sectional study
Source: BMC Med Educ. 2026 May 13;26:1087. doi: 10.1186/s12909-026-09411-4 (PMC13340372; doi:10.1186/s12909-026-09411-4)
Supplement: Supplementary file 2 — Supplementary Material 2. [file 12909_2026_9411_MOESM2_ESM.docx]

**Instructions to Participants**

You are invited to participate in a study assessing pharmacy students’ awareness, perceptions, and intent to pursue postgraduate education in pharmaceutical marketing. Your responses are anonymous and will be used for research purposes only.

**Section A: Demographic Information**

1. **Age:**

- ☐ <18
- ☐ 18–20
- ☐ 21–23
- ☐ 24–26
- ☐ >26

1. **Gender:**

- ☐ Male
- ☐ Female

1. **GPA:**

- ☐ 1.1–2.0
- ☐ 2.1–3.0
- ☐ 3.1–4.0

1. **Marital Status:**

- ☐ Single
- ☐ Married

1. **Nationality:**

- ☐ Saudi
- ☐ Non-Saudi

1. **Satisfaction with your current education:**

- ☐ Very dissatisfied
- ☐ Dissatisfied
- ☐ Neutral
- ☐ Satisfied
- ☐ Very satisfied

**Section B: Awareness of Pharmaceutical Marketing Postgraduate Education**

(Responses: Yes / No)

1. Have you heard about postgraduate programs in pharmaceutical marketing?

- ☐ Yes
- ☐ No

1. Do you have information about career opportunities in pharmaceutical marketing?

- ☐ Yes
- ☐ No

1. Have you received any formal or informal training related to pharmaceutical marketing?

- ☐ Yes
- ☐ No

**Section C: Perceived Importance**

**(Responses: 1 = Strongly Disagree → 5 = Strongly Agree)**

1. A master’s degree in pharmaceutical marketing will improve my career prospects.
2. It will enhance my chances of obtaining leadership or managerial roles.
3. It will improve my professional skills in the pharmaceutical field.
4. It can contribute to improving patient outcomes.
5. It is important for the development of the pharmaceutical industry.

**Section D: Supportive Environment**

**(Responses: 1 = Not Important → 5 = Very Important)**

1. Availability of scholarships
2. Tuition financial support
3. Flexible work schedules
4. Availability of online programs
5. Availability of accelerated programs

**Section E: Intention to Pursue Postgraduate Education**

**(Responses: 1 = Strongly Disagree → 5 = Strongly Agree)**

1. I am interested in pursuing a master’s degree in pharmaceutical marketing.
2. I plan to apply for a postgraduate program in pharmaceutical marketing.
3. I am motivated to continue my studies in this field.
4. I would recommend this field to others.
